# Cultured *ex vivo* human brain tissue maintains cell-type transcriptional identities

**DOI:** 10.1093/braincomms/fcag073

**Published:** 2026-03-09

**Authors:** J P McGinnis, Sai Mallannagari, Maria Camila Guevara, Joshua Ortiz-Guzman, Benjamin D W Belfort, Suyang Bao, Snigdha Srivastava, Maria Morkas, Emily Ji, Kalman A Katlowitz, Angela Addison, Evelyne K Tantry, Carrie A Mohila, Melissa M Blessing, Nisha Gadgil, Samuel G McClugage, David F Bauer, William E Whitehead, Guillermo Aldave, Omar Tanweer, Naser Jaleel, Ali Jalali, Akash J Patel, Sameer A Sheth, Howard L Weiner, Shankar Gopinath, Ganesh Rao, Akdes Serin Harmanci, Daniel Curry, Benjamin R Arenkiel

**Affiliations:** Department of Neurosurgery, Baylor College of Medicine, Houston, TX 77030, USA; Jan and Dan Duncan Neurological Research Institute, Texas Children's Hospital, Houston, TX 77030, USA; Department of Molecular and Human Genetics, Baylor College of Medicine, Houston, TX 77030, USA; Jan and Dan Duncan Neurological Research Institute, Texas Children's Hospital, Houston, TX 77030, USA; Department of Molecular and Human Genetics, Baylor College of Medicine, Houston, TX 77030, USA; Jan and Dan Duncan Neurological Research Institute, Texas Children's Hospital, Houston, TX 77030, USA; Department of Molecular and Human Genetics, Baylor College of Medicine, Houston, TX 77030, USA; Jan and Dan Duncan Neurological Research Institute, Texas Children's Hospital, Houston, TX 77030, USA; Department of Molecular and Human Genetics, Baylor College of Medicine, Houston, TX 77030, USA; Jan and Dan Duncan Neurological Research Institute, Texas Children's Hospital, Houston, TX 77030, USA; Medical Scientist Training Program, Baylor College of Medicine, Houston, TX 77030, USA; Department of Molecular and Human Genetics, Baylor College of Medicine, Houston, TX 77030, USA; Development, Disease Models & Therapeutics Graduate Program, Baylor College of Medicine, Houston, TX 77030, USA; Department of Molecular and Human Genetics, Baylor College of Medicine, Houston, TX 77030, USA; Jan and Dan Duncan Neurological Research Institute, Texas Children's Hospital, Houston, TX 77030, USA; Medical Scientist Training Program, Baylor College of Medicine, Houston, TX 77030, USA; Department of Molecular and Human Genetics, Baylor College of Medicine, Houston, TX 77030, USA; Department of Molecular and Human Genetics, Baylor College of Medicine, Houston, TX 77030, USA; Department of Neurosurgery, Baylor College of Medicine, Houston, TX 77030, USA; Department of Molecular and Human Genetics, Baylor College of Medicine, Houston, TX 77030, USA; Development, Disease Models & Therapeutics Graduate Program, Baylor College of Medicine, Houston, TX 77030, USA; Department of Molecular and Human Genetics, Baylor College of Medicine, Houston, TX 77030, USA; Jan and Dan Duncan Neurological Research Institute, Texas Children's Hospital, Houston, TX 77030, USA; Department of Pathology, Texas Children’s Hospital, Baylor College of Medicine, Houston, TX 77030, USA; Department of Pathology, Texas Children’s Hospital, Baylor College of Medicine, Houston, TX 77030, USA; Department of Neurosurgery, Baylor College of Medicine, Houston, TX 77030, USA; Department of Neurosurgery, Texas Children’s Hospital, Baylor College of Medicine, Houston, TX 77030, USA; Department of Neurosurgery, Baylor College of Medicine, Houston, TX 77030, USA; Department of Neurosurgery, Texas Children’s Hospital, Baylor College of Medicine, Houston, TX 77030, USA; Department of Neurosurgery, Baylor College of Medicine, Houston, TX 77030, USA; Department of Neurosurgery, Texas Children’s Hospital, Baylor College of Medicine, Houston, TX 77030, USA; Department of Neurosurgery, Baylor College of Medicine, Houston, TX 77030, USA; Department of Neurosurgery, Texas Children’s Hospital, Baylor College of Medicine, Houston, TX 77030, USA; Department of Neurosurgery, Baylor College of Medicine, Houston, TX 77030, USA; Department of Neurosurgery, Texas Children’s Hospital, Baylor College of Medicine, Houston, TX 77030, USA; Department of Neurosurgery, Baylor College of Medicine, Houston, TX 77030, USA; Department of Neurosurgery, Baylor College of Medicine, Houston, TX 77030, USA; Department of Neurosurgery, Baylor College of Medicine, Houston, TX 77030, USA; Department of Neurosurgery, Baylor College of Medicine, Houston, TX 77030, USA; Department of Neurosurgery, Baylor College of Medicine, Houston, TX 77030, USA; Department of Neurosurgery, Baylor College of Medicine, Houston, TX 77030, USA; Department of Neurosurgery, Texas Children’s Hospital, Baylor College of Medicine, Houston, TX 77030, USA; Department of Neurosurgery, Baylor College of Medicine, Houston, TX 77030, USA; Department of Neurosurgery, Baylor College of Medicine, Houston, TX 77030, USA; Department of Neurosurgery, Baylor College of Medicine, Houston, TX 77030, USA; Jan and Dan Duncan Neurological Research Institute, Texas Children's Hospital, Houston, TX 77030, USA; Department of Neurosurgery, Baylor College of Medicine, Houston, TX 77030, USA; Department of Neurosurgery, Texas Children’s Hospital, Baylor College of Medicine, Houston, TX 77030, USA; Department of Molecular and Human Genetics, Baylor College of Medicine, Houston, TX 77030, USA; Jan and Dan Duncan Neurological Research Institute, Texas Children's Hospital, Houston, TX 77030, USA

**Keywords:** organotypic slice culture, *ex vivo* human brain, temporal lobectomy, glioblastoma, medulloblastoma

## Abstract

There is growing recognition of the need for human-based models of the nervous system and its diseases. Studies have investigated the electrophysiological properties of neurons in *ex vivo* human brain tissue, but the maintenance of other cell types remains unclear. We therefore used single-nucleus RNA sequencing of six patient samples to determine how well various cell types maintain their transcriptional identities over two weeks in organotypic slice culture (day 0, with 83 501 nuclei, and day 14, with 45 738). For each patient sample (two pediatric temporal lobectomies, one adult frontal cortex, two glioblastomas, and one medulloblastoma), we generated correlations between each day 0 and day 14 cell type using the extent of up- and down-regulation of all significantly variable genes. We found generally high correlations, especially in tumour cells (glioblastoma tumour cells r = 0.81, medulloblastoma tumour cells r = 0.90), with microglia (r = 0.72), oligodendrocytes (r = 0.76), and endothelial cells (r = 0.75). This holds significant promise for this model’s use in both mechanistic studies and therapeutic screens, with its high degree of molecular and cellular relevance to the *in vivo* human brain.

## Teaser

McGinnis *et al*. demonstrate that *ex vivo* human brain tissues maintained in organotypic slice culture preserve the transcriptional identities of major cell types—including tumour and non-tumour populations—over two weeks, establishing the foundation for robust human-based models of the central nervous system for mechanistic studies and therapeutic screening.

## Graphical Abstract

**Graphical Abstract:**
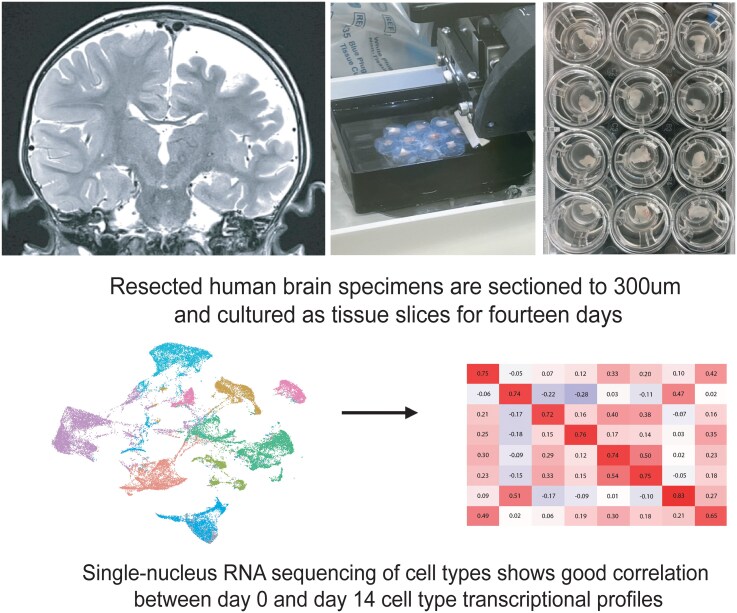

## Introduction

The translational limitations of animal models are widely acknowledged, with growing recognition of the need for models with greater predictive validity for human biology, especially the nervous system.^1-3^ One promising approach is the organotypic brain slice culture, which maintains intact slices of resected human brain tissue in a living state *ex vivo.*^4,5^ Importantly, in order to serve as reliable predictors of the human nervous system’s molecular and cellular behaviour, cultured tissues should ideally maintain the relevant features of the brain’s cell types across a reasonable time in culture.

Several groups have studied the ability of organotypic brain slices to preserve the functional electrophysiological profiles of human neurons.^6-9^ This has further allowed optogenetic manipulation of human neuron firing rates *in vitro.*^10^ However, because many human brain pathologies also involve non-neuronal cell types, if this model is to be useful for mechanistic studies and therapeutic screens, it would ideally maintain the identities of all cell types found within the human brain, normal or diseased.

The purpose of this study was to evaluate how well the transcriptional identities of all cell types found in resected human brain tissue were maintained across two weeks in organotypic (non-dissociated) slice culture, and therefore how well the model in its current state can be expected to represent *in vivo* human brain cell populations. This would also guide the development of the human brain organotypic model, with the goal of maintaining these tissues as close as possible to their *in vivo* state. We used single-nucleus RNA sequencing to compare the transcriptional identities of cell types in brain slices that were cultured for two weeks (day 14) with tissue from the same patients frozen immediately after resection (day 0).^11,12^ We chose day 14 as a time point since that duration would permit basic molecular interventions but ideally still retain important molecular and cellular features. We used six patient samples: three tissues with near-normal cell types (two pediatric temporal lobe cortex samples resected for temporal lobe epilepsy and one adult frontal cortex sample overlying a deeper insular tumour), two IDH wild-type glioblastoma tumour samples (one treatment-naïve, the other having undergone a prior resection and chemotherapy/radiation), and one SHH-type medulloblastoma sample. These were chosen because they offered a reasonable representation of both near-normal and neoplastic tissue types for which effective therapies are still being sought.

## Materials and methods

### Human brain tissue acquisition and culture

Patients (one female, five males, Table 1) were consented (Baylor College of Medicine IRB H-51865) for neurosurgical specimens not needed for pathologic diagnosis. Specimens were transported in ice-cold, pre-carbogenated (>20 min, 95%/5% O2/CO2) NMDG-aCSF, following published protocols.^5^ Larger tissue pieces were sectioned to ∼1 cm pieces, placed into a retractable tube, and molten agarose (Sigma, A2576) was poured over top. Tissues were affixed and sectioned to 300 µm on a Leica VT1200 vibratome (housed within a biosafety cabinet to attempt to improve sterility) in cold aCSF that was continuously bubbled with carbogen. We then moved the slices to room temperature aCSF, bubbling with carbogen, for 15–30 min, after which the slices were gently plated onto membrane inserts (CellTreat, #230617) overlying 600 µL of organotypic slice culture media (OSCM). OSCM was made as previously described,^8^ except that heparin was not used, and the antibiotic-antimycotic (Gibco 15240–062) concentration was increased to 2%. Culture plates were kept at 37 °C, 95% humidity, and 5% CO2 and underwent daily media changes. At the time of collection, four to eight slices were flash frozen together and stored in −80 °C.

**Table 1:** Tissue sources with anatomic location, pathology, and age

Tissue	Anatomic source, pathology	Age
**A**	Temporal lobe cortex, epilepsy, dysplastic hemisphere with migrational abnormalities	6 years old, F
**B**	Temporal lobe cortex, epilepsy, Sturge Weber syndrome	2 years old, M
**C**	Frontal opercular cortex overlying an insular glioma	60 years old, M
**D**	Newly diagnosed IDH wild-type glioblastoma	86 years old, M
**E**	Recurrent IDH wild-type glioblastoma	65 years old, M
**F**	Recurrent SHH medulloblastoma	20 years old, M

### Single-nucleus isolation, library preparation, and sequencing

Frozen tissue was dissociated using the Miltenyi Biotec GentleMACS nuclei isolation protocol, ‘nuclei isolation’ program on a GentleMACS Octo Dissociator, followed by two filtration (70 µm, 30 µm) and centrifugation steps. Nuclei were FACS sorted (DAPI gating) on a Sony MA900 within the Baylor College of Medicine Cytometry Core, with a sorting target of 200 000 nuclei when possible. Due to the smaller tissue input of day 14 samples compared with day 0 samples, it was often the case that fewer nuclei were sorted. Single-cell gene expression libraries were prepared according to the Chromium Single Cell Gene Expression 3’v3.1 instruction (10 × Genomics) by the Baylor College of Medicine Single Cell Core. Barcoded cDNA libraries were submitted for Illumina NovaSeq 2 × 150 (paired end) sequencing (Azenta/Genewiz), targeting ∼1 billion reads per sample, which in preliminary studies had yielded approximately ∼50 000 reads per cell on average.

### Cell type annotation

The 10X Genomics scRNA-seq data were processed using Cell Ranger Count v9.0.1 with the GRCh38.p13 genome. The raw feature gene-cell count matrix was downloaded per sample and processed through CellBender on Ubuntu, trained on 150 complete passes. We then performed the standard Seurat clustering workflow on each sample separately. For each patient sample (at day 0 and day 14 timepoints), a Seurat object was created from raw counts. Cells were retained during quality control if they satisfied the criteria for gene complexity (minimum of 600 detected genes per cell), library size (minimum of 1000 UMIs per cell), and mitochondrial RNA (less than 8% of total genes). Upper outlier caps were computed per sample using the standard 1.5×IQR rule. Each QC-filtered Seurat object was normalized and variance stabilized with SCTransform (per sample) using glmGamPoi and regressing out the mitochondrial fraction. Integration of all patient samples and timepoints followed Seurat’s SCT pipeline: 3000 integration features were selected, SCT objects prepared, PCA run per object on the integration features, integration anchors were found with reciprocal PCA, and data was integrated.

PCA was performed on the scaled and integrated object. The principal components (PC) were ranked based on the percentage of variance. The elbow was observed around PC30; therefore, the top 30 PCs were used for graph construction, clustering, and UMAP generation.

### Near-normal analysis

For the near-normal samples, a reference matrix was built from the Allen Institute’s 10X transcriptomic data collected from the human middle temporal gyrus (MTG). Fine labels were collapsed into broad classes: Astrocytes, Endothelial Cells, GABAergic Neurons, Glutamatergic Neurons, Microglia, Oligodendrocytes, Oligodendrocyte Precursor Cells (OPCs), and Vascular Leptomeningeal Cells (VLMC). For each gene and fine label entry, median expressions were averaged within each broad class to yield a gene × broad cell types expression matrix. The query data were log-normalized, intersected to common genes with reference, and SingleR was then run to assign per-cell labels to the Seurat object.

### Tumour cells and SCRAM analysis

We ran our aggregated glioblastoma Seurat objects through the SCRAM classifier, the details of which have been previously published.^14^ SCRAM is a novel computational classifier trained on both normal and tumour cell types. We aggregated cell type assignments into general categories.

We carried out an analysis of the medulloblastoma Seurat object using the medulloblastoma-tuned SCRAM protocol.

### Log2 fold change calculations

Using the 10 ampa#thinsp; Loupe browser, we first selected only the day 0 cell types and generated differential expression profiles for each cell type compared to all other day 0 cells (exact negative binomial test [Yu, Huber, & Vitek, 2013]). We then repeated this process, substituting a single day 14 cell type in for its day 0 counterpart to generate that cell type’s log2 fold change relative to all other cell types at day 0. We removed genes with minimal expression (average occurrence < 1 count across the dataset) and downloaded CSV files of all significantly variable genes (Benjamini-Hochberg correction, adjusted *P*-value < 0.05). Custom R code was written to split the CSVs into their individual cell types that contained gene names, average expression level, log2 fold change, and *P*-value. All significantly variable genes found for a patient sample in at least one cell type were compiled, and that gene list was used to compare day 0 to day 14 cell types, regardless of whether a gene was significantly variable for the given cell type being compared. The correlation between the log2 fold changes for each cell type was calculated using custom R code. Custom R code was used on the cell type CSVs to generate correlation matrices using ggplot. (All code available on GitHub, github.com/jpmcginnis/HumanAAVProject.) Graphs were created in Graphpad Prism 10.

### Differential expression analysis

Having identified the significantly differentially expressed genes for day 14 compared to day 0 for each cell type, we performed gene set enrichment analysis. Using the Hallmark gene set, we identified broad pathways that showed normalized enrichment scores > 1.5 (absolute value) and adjusted *P*-value < 0.05. These were plotted as heatmaps for each cell type across our three sets of tissues.

## Statistical analysis

Data are presented as mean ± SEM unless otherwise indicated. For single-nucleus RNA sequencing analysis, differential expression analysis was performed with Benjamini-Hochberg correction for multiple comparisons. Significantly variable or significantly differentially expressed genes were included if the adjusted *P*-value < 0.05.

## Results

**Figure 1:**
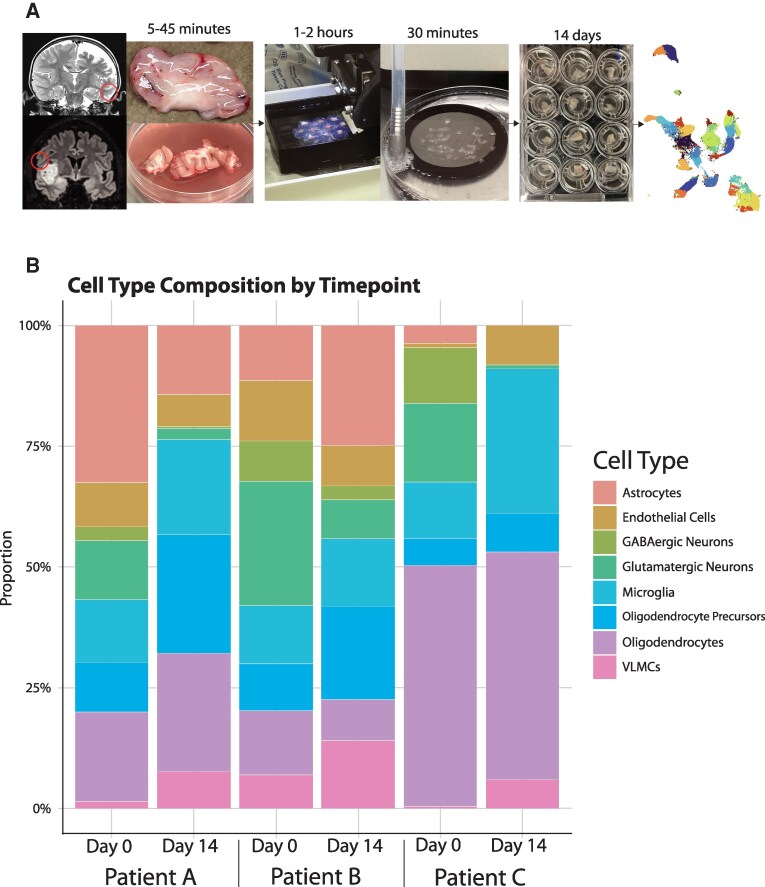
**The human brain organotypic slice culture model is feasible and shows conservation of most cell types across time. A,** Workflow of the human brain organotypic slice culture model, from MRI to tissue sectioning and culture plates to single-nucleus RNA sequencing. **B**, Bar graphs showing the relative proportions of identified cell types in day 0 and day 14 samples for each patient tissue (tissue A 2507 and 3073 cells, tissue B 13 005 and 10 969 cells, tissue C 6642 and 2955 cells). Cell types were identified using canonical marker gene expression from published single-cell and single-nucleus RNA sequencing data and annotated using SingleR.

Patients who planned to undergo resective surgeries consented to any removed tissue that was not needed for pathologic diagnosis. Tissue samples were collected from the OR or pathology within 45 min of resection, transported in ice-cold pre-carbogenated NMDG-aCSF, sectioned to 300 um, and either flash frozen in liquid nitrogen (day 0) or plated on membrane inserts overlying OSCM, where they underwent daily media changes for two weeks (day 14) (Fig. 1A, Table 1).

### Near-normal tissue composition

Single-nucleus RNA sequencing libraries were prepared, sequenced, and aligned to the human reference genome (GRCh38), and each patient sample’s day 0 and day 14 data were integrated and plotted in UMAP space. Near-normal tissue types (patients A-C) were annotated using the SingleR package to assign cell types based on the Allen Institute Human MTG—10X atlas and then collapsed into general categories (for original cell type assignments, see Supplementary Fig. 1A). We examined typical marker genes’ expression to confirm the appropriate assignments of cell types (Supplementary Fig. 1B). We first plotted the proportions for each patient’s day 0 and day 14 samples (Fig. 1B).^13^ In most tissues, microglia, oligodendrocytes, oligodendrocyte precursor cells, and endothelial cells all generally remained stable, while astrocytes were more variable (and lost in the adult sample, patient C). The share of neurons at day 14 was lower in all samples than in day 0 tissues.

### Gene expression preservation in near-normal cell types

**Figure 2:**
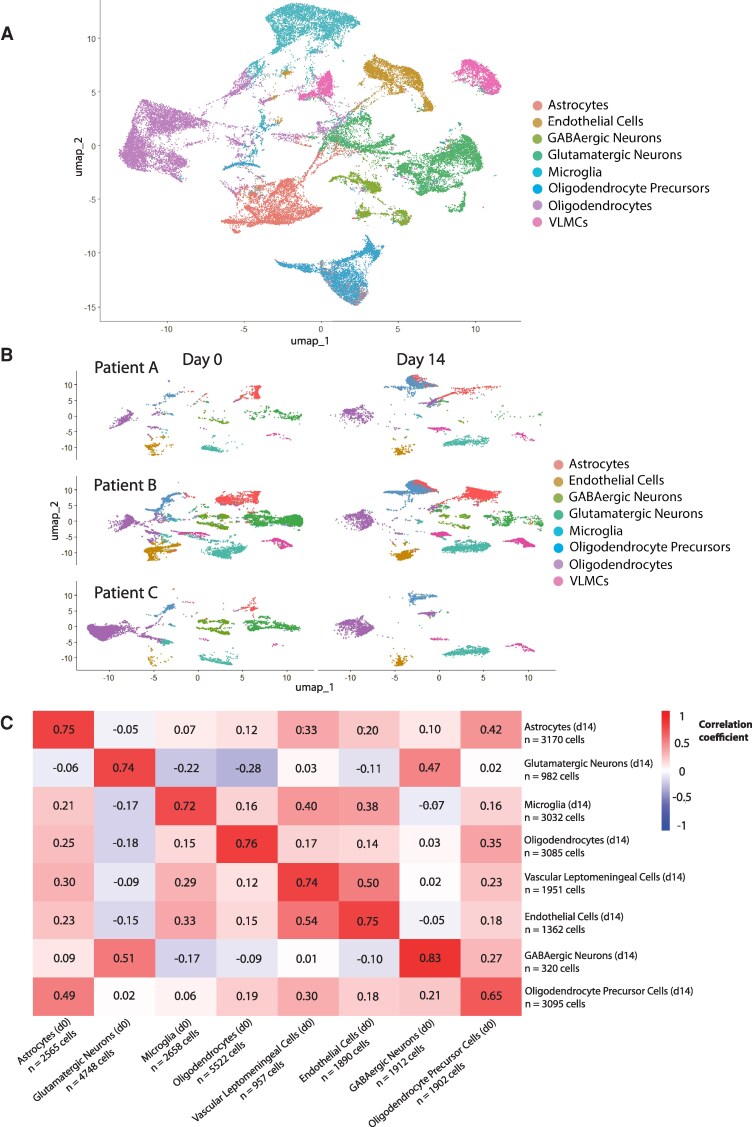
**Single-nucleus RNA sequencing shows retained cell type transcriptional identities in tissues with more normal cell types.** A, Integrated UMAP projections of Day 0 and Day 14 cells for tissues A, B, and C. Each point represents a single cell. **B**, Split UMAPs colour-coded by cell type for day 0 and day 14 cells for tissues A, B, and C. Day 0 and day 14 cell types generally cluster together, though there are some differences, e.g. tissue C’s neurons in day 0 and day 14. Cell types assigned using published marker genes (see methods) (tissue A 2507 and 3073 cells, tissue B 13 005 and 10 969 cells, tissue C 6642 and 2955 cells). **C**, Pearson correlation matrices comparing the log2 fold change of a given cell type at day 14 to cell types at day 0. Log2 fold changes were generated by comparing a given cell type at day 0 to the rest of the day 0 cells, and given cell types at day 14 to the rest of the day 0 cells (in essence, seeing how well the cell-type-specific properties of its transcriptional profile are preserved with time). Higher correlations at intersections of cell types indicate that the unique transcriptional profile differentiating that cell type from other cell types at day 0 is better preserved at day 14. (Heatmap scale bar colours indicate the Pearson correlation coefficient value.)

We asked whether near-normal cell types from day 14 would be similar enough to day 0 that they would tend to cluster together, or whether time point or patient effects would predominate. We observed that cell types did in fact cluster together almost universally across samples, including samples from different patients and time points (Fig. 2A and B).

We next evaluated how well the distinctive gene expression profile of each present cell type was maintained over fourteen days in culture. We first aggregated all three near-normal patient samples. We used the day 0 samples to generate a patient-specific list of genes that were significantly up- or down-regulated (log2 fold changes, adjusted *P*-value < 0.05). We then repeated that analysis but substituted a given day 14 cell type in place of its day 0 counterpart, generating its gene expression profile compared to other day 0 cell types. We calculated the correlation between a cell type’s log2 fold changes at day 0 and day 14 (compared to all other day 0 cell types). This allowed us to ask whether a cell type's genes that tended to be up- or down-regulated at day 0 relative to other cell types also tended to be up- or down-regulated after fourteen days in culture—a higher correlation indicates that a cell type’s gene expression profile was better maintained over two weeks in culture.

In general, cell types showed strong correlation between their day 0 and day 14 transcriptional profiles, with all cell types showing correlation coefficients between 0.65 (oligodendrocyte precursor cells) and 0.83 (GABAergic neurons, though these were absent in the patient C day 14 sample) (Fig. 2C).

### Tumour tissue composition

**Figure 3:**
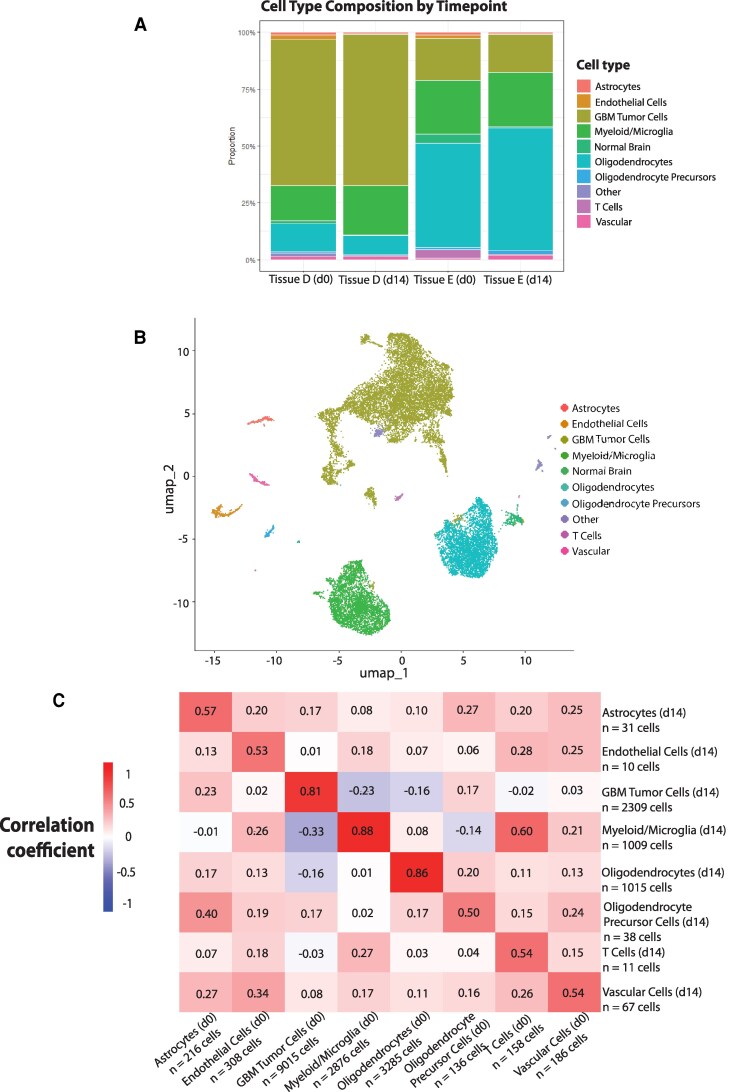
**Cells within glioblastoma tumour tissues retain their unique transcriptional profile after two weeks in culture. A**, Cell type composition for the two IDH-wild type glioblastoma samples, comparing day 0 to day 14 single-nucleus RNA sequencing results (tissue D 12 925 cells and 3109 cells, tissue E 3707 cells and 1393 cells). **B,** Combined UMAPs for glioblastoma tissues D and E, colour-coded by cell types. **C**, Correlation matrices (Pearson) comparing combined day 0 to combined day 14 cell types’ transcriptional profiles for tissues D (IDH wild-type glioblastoma) and E (IDH wild-type glioblastoma).

**Figure 4:**
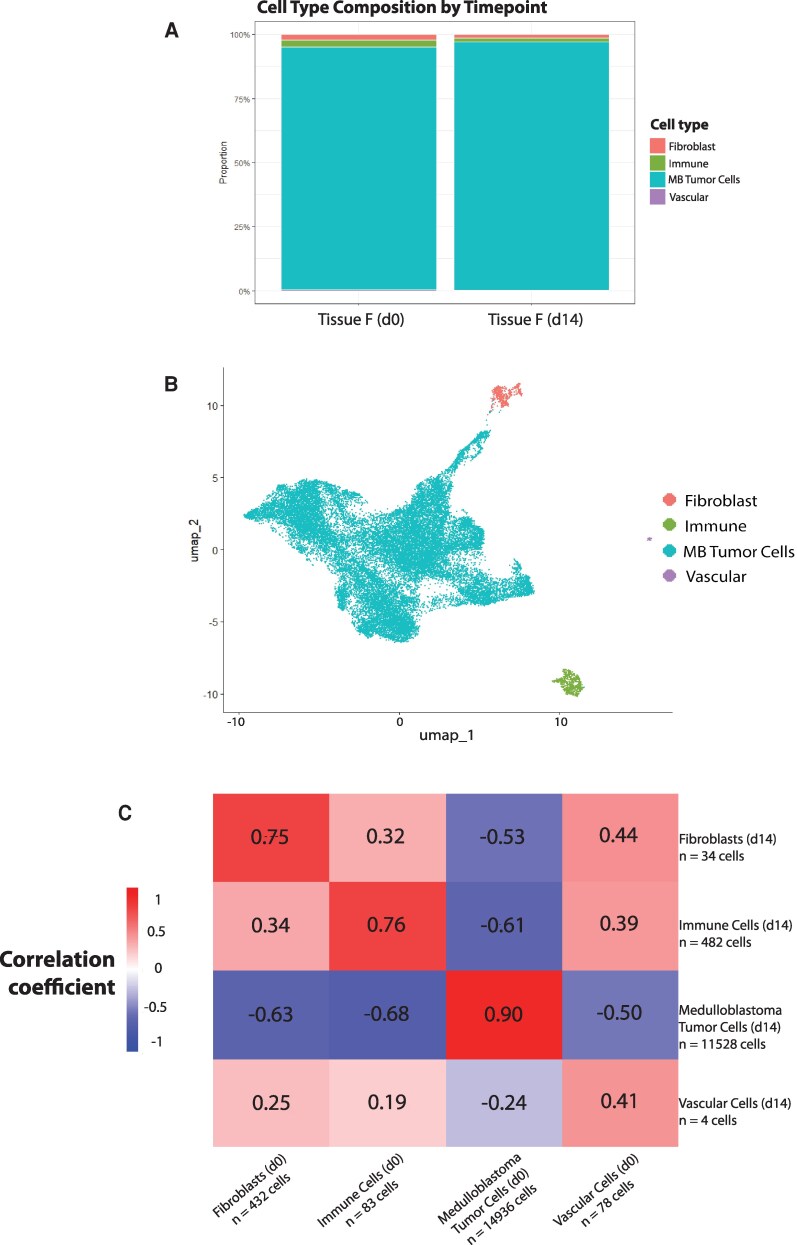
**Medulloblastoma tumour cells retain their unique transcriptional profile after two weeks in culture. A**, Cell type composition for the medulloblastoma sample, comparing day 0 to day 14 single-nucleus RNA sequencing results (day 015 529 cells, day 1 412 048 cells). **B,** Combined UMAP for medulloblastoma tissue (tissue F) colour-coded by cell types. **C,** Correlation matrix (Pearson) comparing day 0 to day 14 cell types’ transcriptional profiles for the medulloblastoma sample. (Heatmap scale bar colours indicate the Pearson correlation coefficient value.)

Because tumour cells may respond differently to *ex vivo* culture conditions, we carried out similar analyses for two IDH wild-type glioblastoma samples and one medulloblastoma sample. Cell types were assigned using the recently published SCRAM classifier, a novel computational pipeline to assign tumour cell types.^14^ We found that the proportions of glioblastoma (GBM) tumour cells remained largely stable across fourteen days in culture, as did other tumour-associated cell types (Fig. 3A and B). For patient D, a newly diagnosed glioblastoma naive to treatment, tumour cells were the predominant cell type, whereas for patient E, a recurrence that was previously treated with temozolomide and radiation, tumour cells were present but were a minority of the cells (Fig. 3A and B). In our medulloblastoma sample (using the medulloblastoma-focused SCRAM tool), the vast majority of cells were tumour cells, as expected for a less infiltrative tumour (Fig. 4A and B).

### Gene expression preservation in tumour cell types

We then sought to determine whether malignant and tumour-associated cell transcriptional profiles would also be maintained over two weeks in culture. Many studies have looked at the preservation of marker genes in short-term explant, organoids, or xenograft models, but studies assessing the global transcriptional preservation of each tumour-associated cell typeare lacking.

Having aggregated our two glioblastoma patient samples, and with cell types assigned by SCRAM, we found the highest transcriptional preservation in myeloid/microglia (r = 0.88), oligodendrocytes (0.86), and glioblastoma tumour cells (0.81). All other cell types—astrocytes, endothelial cells, OPCs, T cells, and other vascular cells—showed moderate correlations, in the 0.5 to 0.6 range (Fig. 3C).

For our medulloblastoma patient sample, tumour cells showed the best preservation of transcriptional profile across fourteen days (r = 0.90), followed by immune cells (0.76) and fibroblasts (0.75), with lower correlation seen in vascular cells (0.41) (Fig. 4C).

In sum, tumour cells show quite strong preservation of their global transcriptional profile across fourteen days in *ex vivo* slice culture, with other cell types showing moderate to very strong preservation.

### Differential gene expression between day 0 and day 14 tissue

To complement the assessment of similarity between each cell type at day 0 and day 14, we identified the differentially expressed genes between day 14 and day 0 samples for each cell type. Our goal was to identify any broadly shared programs across multiple cell types that could indicate pressures of *ex vivo* culture, with the rationale of developing culture conditions that better preserving ‘day 0’ transcriptional profiles across time.

We used the MSigDB Hallmarks gene set to summarize our differential expression data.^15^ For the near-normal samples, few signatures were broadly shared (androgen response, KRAS signalling downregulation, TNFA signalling, and UV response were all downregulated across several cell types; oxidative phosphorylation was upregulated across several cell types), suggesting no systematic perturbations over fourteen days in culture (Supplementary Fig. 2). Glutamatergic neurons and oligodendrocytes showed upregulation across most signatures, whereas endothelial cells and GABAergic neurons showed more consistent downregulation. Other cell types showed fewer or mixed shifts in hallmark signatures.

For our glioblastoma samples, GBM tumour cells showed consistent upregulation across a range of signatures, including DNA repair, interferon responses, MYC targets, oxidative phosphorylation, p53 signalling, and unfolded protein responses (Supplementary Fig. 3). Microglia/myeloid and OPCs showed relatively few programs with significant changes, with oligodendrocytes and vascular cells also upregulating many signatures. No hallmark signature showed directionally similar changes in more than three cell types.

Medulloblastoma tumour cells showed fewer significant shifts, but those that were significant were upregulated, including angiogenesis, epithelial-to-mesenchymal transition, glycolysis, and inflammatory responses (Supplementary Fig. 4). Immune cells showed broader upregulation across hallmarks, while fibroblast shifts were mixed.

Across all these cell types, common upregulated hallmarks often include stress and inflammatory responses (JAK/STAT, interferons, inflammatory responses, unfolded protein responses) and metabolism changes (glycolysis, oxidative phosphorylation), suggesting that future ex vivo culture work should focus on mitigating the nutrient stress of these tissue slices. A certain number of ‘wound healing’ signatures are likely inevitable with tissue obtained from surgeries and sliced to thin sections in culture, but better preserving all cell types in their ‘day 0’ states will be necessary for the optimal predictive validity that *ex vivo* human tissue promises.

## Discussion

The human brain organotypic slice model, which contains all cell types of a resected human brain specimen and its intact local architecture, promises a higher degree of molecular and cellular relevance to *in vivo* human brain tissue. Our goal was to determine how well gene expression profiles in this *ex vivo* human brain tissue are preserved over two weeks in culture, while also establishing a baseline so that future improvements in culture conditions can be evaluated by this transcriptional fidelity (in the form of higher correlation coefficients to day 0, or immediately resected, human brain). Across six patient samples, comprising 83 501 nuclei from day 0 samples and 45 738 nuclei from day 14 samples, tumor cells (from both glioblastoma and medulloblastoma), microglia, endothelial cells, oligodendrocytes, and inhibitory neurons strongly maintained their transcriptional profiles over two weeks in culture (Fig. 3C, Fig. 4C), while astrocytes and excitatory neurons (when present) showed moderate but reasonable preservation. It is important to note that this data is from just six patient samples, comprising three distinct types of brain tissues obtained during neurosurgical resections. Further work is needed to corroborate these findings of generally preserved transcriptional profiles, determine media and culture conditions that can improve preservation, and ascertain whether preservation is seen in other brain pathologies.

It is notable that there are substantially fewer neurons at day 14 in the near-normal samples. With the caveat that single-nucleus RNA sequencing relies on sorting for viable nuclei, and accurate proportions of cells may be different from those reflected by sequencing, we do suspect there is neuronal loss over time that will need to be addressed. This could be due to their susceptibility to hypoxia during tissue collection and transport, or to the use of defined culture media, which is not a perfect substitute for *in vivo* conditions. Tumour cells may be able to resist this in part due to their ability to tolerate relative hypoxia and suboptimal growth conditions. Some electrophysiological studies have shown better outcomes in characterising and culturing neurons using human CSF as a culture medium.^5^ It may also be the case that shorter durations in culture (e.g. 7 days) may show better neuronal preservation and yet be sufficiently long for certain experiments.

One argument frequently voiced against the use of human samples is the variability intrinsic to each patient. It is true that patients’ diseases are more variable than cell culture lines, organoid clones, or inbred mouse strains. It is also true, however, that human variability is an obstacle faced by every therapy that makes it to clinical trials,^8^ and its introduction into preclinical stages may prove valuable in guiding decisions surrounding advancement into clinical trials.

Because of the vast differences between the human brain and animal models, the use of human-based models is vital but currently under-utilized. Not all labs will have ready access to *ex vivo* human brain tissue; those that do have the obligation to collaborate widely to address the most pressing questions that these models can answer. Given the relatively low threshold for successfully culturing human brain tissue and the benefits of models with greater predictive validity, we should more broadly adopt this model across a wide range of problems and diseases.

## Supplementary Material

fcag073_Supplementary_Data

## Data Availability

All RNAseq data in this paper are available on GEO and can be obtained immediately by request from the corresponding authors. All code used for this work is available at https://github.com/jpmcginnis/HumanAAVProject.
