# Supplementary material for: Cultured ex vivo human brain tissue maintains cell-type transcriptional identities
Source: Brain Commun. 2026 Mar 9;8(2):fcag073. doi: 10.1093/braincomms/fcag073 (PMC13056709; doi:10.1093/braincomms/fcag073)
Supplement: fcag073_Supplementary_Data [file fcag073_supplementary_data.pdf]

A

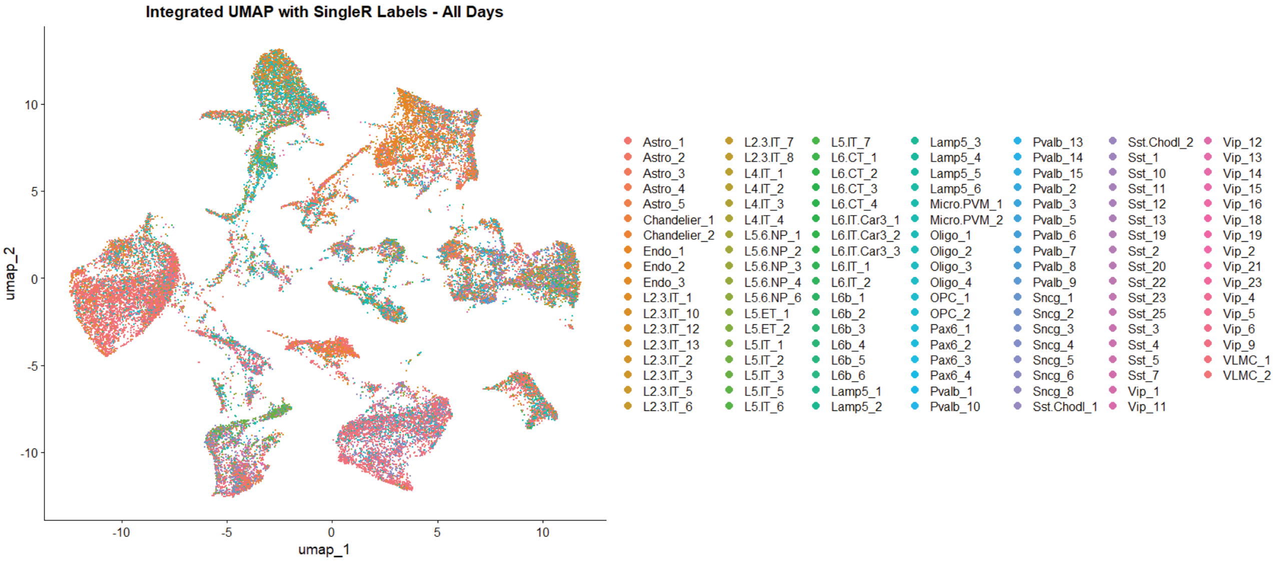

Integrated UMAP with SingleR Labels - All Days
umap_2
umap_1
10
5
0
-5
-10
-10
-5
0
5
10
Astro_1
Astro_2
Astro_3
Astro_4
Astro_5
Chandelier_1
Chandelier_2
Endo_1
Endo_2
Endo_3
L2.3.IT_1
L2.3.IT_10
L2.3.IT_12
L2.3.IT_13
L2.3.IT_2
L2.3.IT_3
L2.3.IT_5
L2.3.IT_6
L2.3.IT_7
L2.3.IT_8
L4.IT_1
L4.IT_2
L4.IT_3
L4.IT_4
L5.6.NP_1
L5.6.NP_2
L5.6.NP_3
L5.6.NP_4
L5.6.NP_6
L5.ET_1
L5.ET_2
L5.IT_1
L5.IT_2
L5.IT_3
L5.IT_5
L5.IT_6
L5.IT_7
L6.CT_1
L6.CT_2
L6.CT_3
L6.CT_4
L6.IT.Car3_1
L6.IT.Car3_2
L6.IT.Car3_3
L6.IT_1
L6.IT_2
L6b_1
L6b_2
L6b_3
L6b_4
L6b_5
L6b_6
Lamp5_1
Lamp5_2
Lamp5_3
Lamp5_4
Lamp5_5
Lamp5_6
Micro.PVM_1
Micro.PVM_2
Oligo_1
Oligo_2
Oligo_3
Oligo_4
OPC_1
OPC_2
Pax6_1
Pax6_2
Pax6_3
Pax6_4
Pvalb_1
Pvalb_10
Pvalb_13
Pvalb_14
Pvalb_15
Pvalb_2
Pvalb_3
Pvalb_5
Pvalb_6
Pvalb_7
Pvalb_8
Pvalb_9
Sncg_1
Sncg_2
Sncg_3
Sncg_4
Sncg_5
Sncg_6
Sncg_8
Sst.Chodl_1
Sst.Chodl_2
Sst_1
Sst_10
Sst_11
Sst_12
Sst_13
Sst_19
Sst_2
Sst_20
Sst_22
Sst_23
Sst_25
Sst_3
Sst_4
Sst_5
Sst_7
Vip_1
Vip_11
Vip_12
Vip_13
Vip_14
Vip_15
Vip_16
Vip_18
Vip_19
Vip_2
Vip_21
Vip_23
Vip_4
Vip_5
Vip_6
Vip_9
VLMC_1
VLMC_2

B

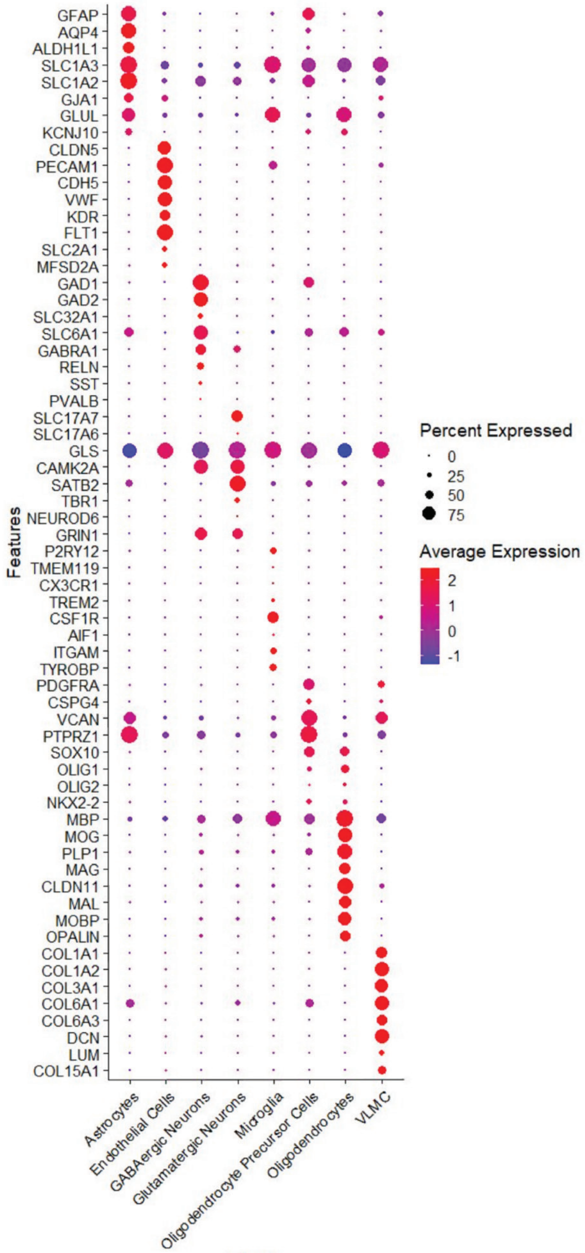

GFAP
AQP4
ALDH1L1
SLC1A3
SLC1A2
GJA1
GLUL
KCNJ10
CLDN5
PECAM1
CDH5
VWF
KDR
FLT1
SLC2A1
MFSD2A
GAD1
GAD2
SLC32A1
SLC6A1
GABRA1
RELN
SST
PVALB
SLC17A7
SLC17A6
GLS
CAMK2A
SATB2
TBR1
NEUROD6
GRIN1
P2RY12
TMEM119
CX3CR1
TREM2
CSF1R
AIF1
ITGAM
TYROBP
PDGFRA
CSPG4
VCAN
PTPRZ1
SOX10
OLIG1
OLIG2
NKX2-2
MBP
MOG
PLP1
MAG
CLDN11
MAL
MOBP
OPALIN
COL1A1
COL1A2
COL3A1
COL6A1
COL6A3
DCN
LUM
COL15A1
Features
Astrocytes
Endothelial Cells
GABAergic Neurons
Glutamatergic Neurons
Microglia
Oligodendrocyte Precursor Cells
Oligodendrocytes
VLMC
Percent Expressed
0
25
50
75
Average Expression
2
1
0
-1

Supplementary Figure 1. SingleR cluster labeling and marker genes. A. SingleR assigned cell types using the Allen Institute 10X Middle Temporal Gyrus dataset to assign cell types, which were then collapsed into the broader categories seen in Figures 1 and 2. B. Dot plot of marker genes identified as highly upregulated in each cluster, demonstrating the validity of the SingleR assignments. The size of dot reflects the percent of all cells of that cell type expressing the gene, with the color (red for greater expression) indicating the extent of expression within those cells.
(VLMC = vascular leptomeningeal cells)

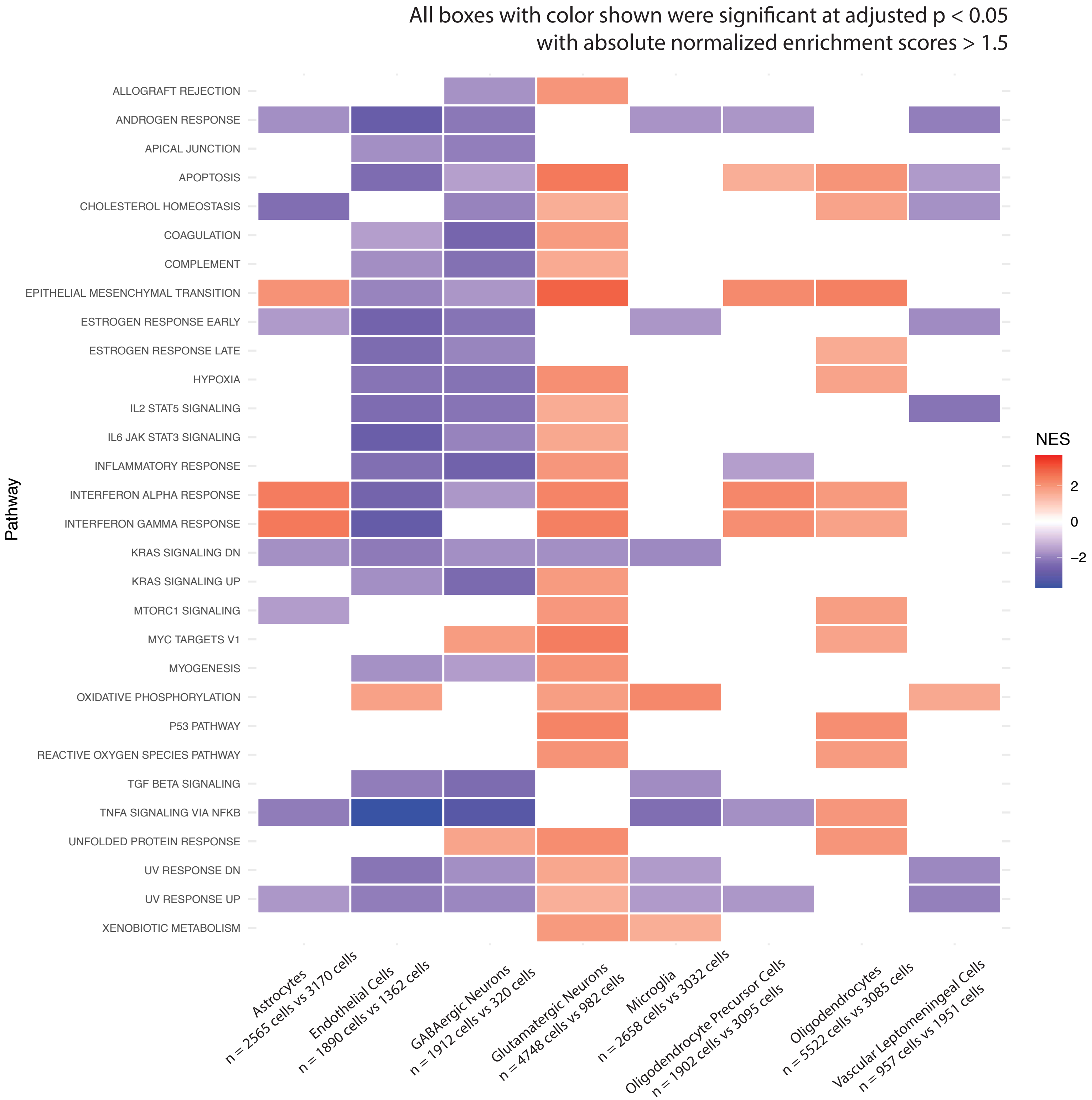
All boxes with color shown were significant at adjusted p < 0.05
with absolute normalized enrichment scores > 1.5
Pathway
ALLOGRAFT REJECTION
ANDROGEN RESPONSE
APICAL JUNCTION
APOPTOSIS
CHOLESTEROL HOMEOSTASIS
COAGULATION
COMPLEMENT
EPITHELIAL MESENCHYMAL TRANSITION
ESTROGEN RESPONSE EARLY
ESTROGEN RESPONSE LATE
HYPOXIA
IL2 STAT5 SIGNALING
IL6 JAK STAT3 SIGNALING
INFLAMMATORY RESPONSE
INTERFERON ALPHA RESPONSE
INTERFERON GAMMA RESPONSE
KRAS SIGNALING DN
KRAS SIGNALING UP
MTORC1 SIGNALING
MYC TARGETS V1
MYOGENESIS
OXIDATIVE PHOSPHORYLATION
P53 PATHWAY
REACTIVE OXYGEN SPECIES PATHWAY
TGF BETA SIGNALING
TNFA SIGNALING VIA NFKB
UNFOLDED PROTEIN RESPONSE
UV RESPONSE DN
UV RESPONSE UP
XENOBIOTIC METABOLISM
NES
2
0
−2
Astrocytes
n = 2565 cells vs 3170 cells
Endothelial Cells
n = 1890 cells vs 1362 cells
GABAergic Neurons
n = 1912 cells vs 320 cells
Glutamatergic Neurons
n = 4748 cells vs 982 cells
Microglia
n = 2658 cells vs 3032 cells
Oligodendrocyte Precursor Cells
n = 1902 cells vs 3095 cells
Oligodendrocytes
n = 5522 cells vs 3085 cells
Vascular Leptomeningeal Cells
n = 957 cells vs 1951 cells

Supplementary Figure 2. MSigDB Hallmark analysis for aggregated near-normal tissues. Heatmap of up- or down-regulated MSigDB Hallmarks across cell types identified in the three aggregated near-normal samples, comparing day 14 cell type transcriptional profiles to day 0 transcriptional profiles. Normalized enrichment scores were generated, along with permutation-based adjusted p values for each pathway by cell type. Cell types that showed significant (Benjamini-Hochberg adjusted p values < 0.05) absolute normalized enrichment values > 1.5 were displayed, with the heatmap color corresponding to the normalized enrichment scores seen in the legend. Nonsignificant pathways are left blank. Number of cells in each cell type for day 0 and day 14 listed at bottom below cell type name. (NES = normalized enrichment score, VLMC = vascular leptomeningeal cells, MYC targets V1 = MYC targets, variant 1, DN = down)

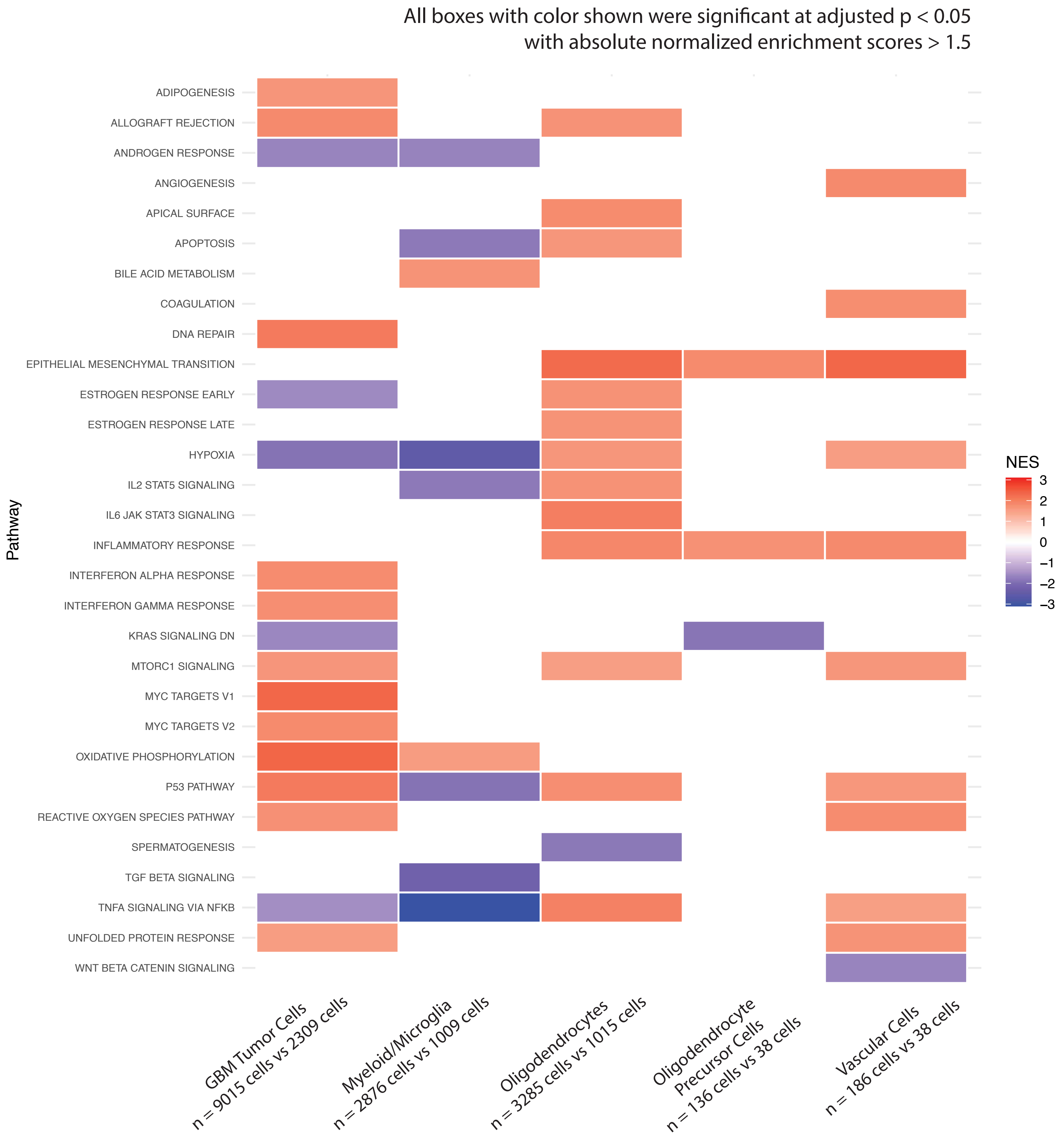
All boxes with color shown were significant at adjusted p < 0.05
with absolute normalized enrichment scores > 1.5
Pathway
ADIPOGENESIS
ALLOGRAFT REJECTION
ANDROGEN RESPONSE
ANGIOGENESIS
APICAL SURFACE
APOPTOSIS
BILE ACID METABOLISM
COAGULATION
DNA REPAIR
EPITHELIAL MESENCHYMAL TRANSITION
ESTROGEN RESPONSE EARLY
ESTROGEN RESPONSE LATE
HYPOXIA
IL2 STAT5 SIGNALING
IL6 JAK STAT3 SIGNALING
INFLAMMATORY RESPONSE
INTERFERON ALPHA RESPONSE
INTERFERON GAMMA RESPONSE
KRAS SIGNALING DN
MTORC1 SIGNALING
MYC TARGETS V1
MYC TARGETS V2
OXIDATIVE PHOSPHORYLATION
P53 PATHWAY
REACTIVE OXYGEN SPECIES PATHWAY
SPERMATOGENESIS
TGF BETA SIGNALING
TNFA SIGNALING VIA NFKB
UNFOLDED PROTEIN RESPONSE
WNT BETA CATENIN SIGNALING
GBM Tumor Cells
n = 9015 cells vs 2309 cells
Myeloid/Microglia
n = 2876 cells vs 1009 cells
Oligodendrocytes
n = 3285 cells vs 1015 cells
Oligodendrocyte
Precursor Cells
n = 136 cells vs 38 cells
Vascular Cells
n = 186 cells vs 38 cells
NES
3
2
1
0
−1
−2
−3

Supplementary Figure 3. MSigDB Hallmark analysis for aggregated glioblastoma tissues. Heatmap of up- or down-regulated MSigDB Hallmarks across cell types identified in the two aggregated glioblastoma samples, comparing day 14 cell type transcriptional profiles to day 0 transcriptional profiles. Normalized enrichment scores were generated, along with permutation-based adjusted p values for each pathway by cell type. Cell types that showed significant (Benjamini-Hochberg adjusted p values < 0.05) absolute normalized enrichment values > 1.5 were displayed, with the heatmap color corresponding to the normalized enrichment scores seen in the legend. Nonsignificant pathways are left blank. Number of cells in each cell type for day 0 and day 14 listed at bottom below cell type name. (NES = normalized enrichment score, OPCs = oligodendrocyte precursor cells, MYC targets V1 = MYC targets, variant 1, MYC targets V2 = MYC targets, variant 2)

All boxes with color shown were significant at adjusted $p < 0.05$
with absolute normalized enrichment scores > 1.5

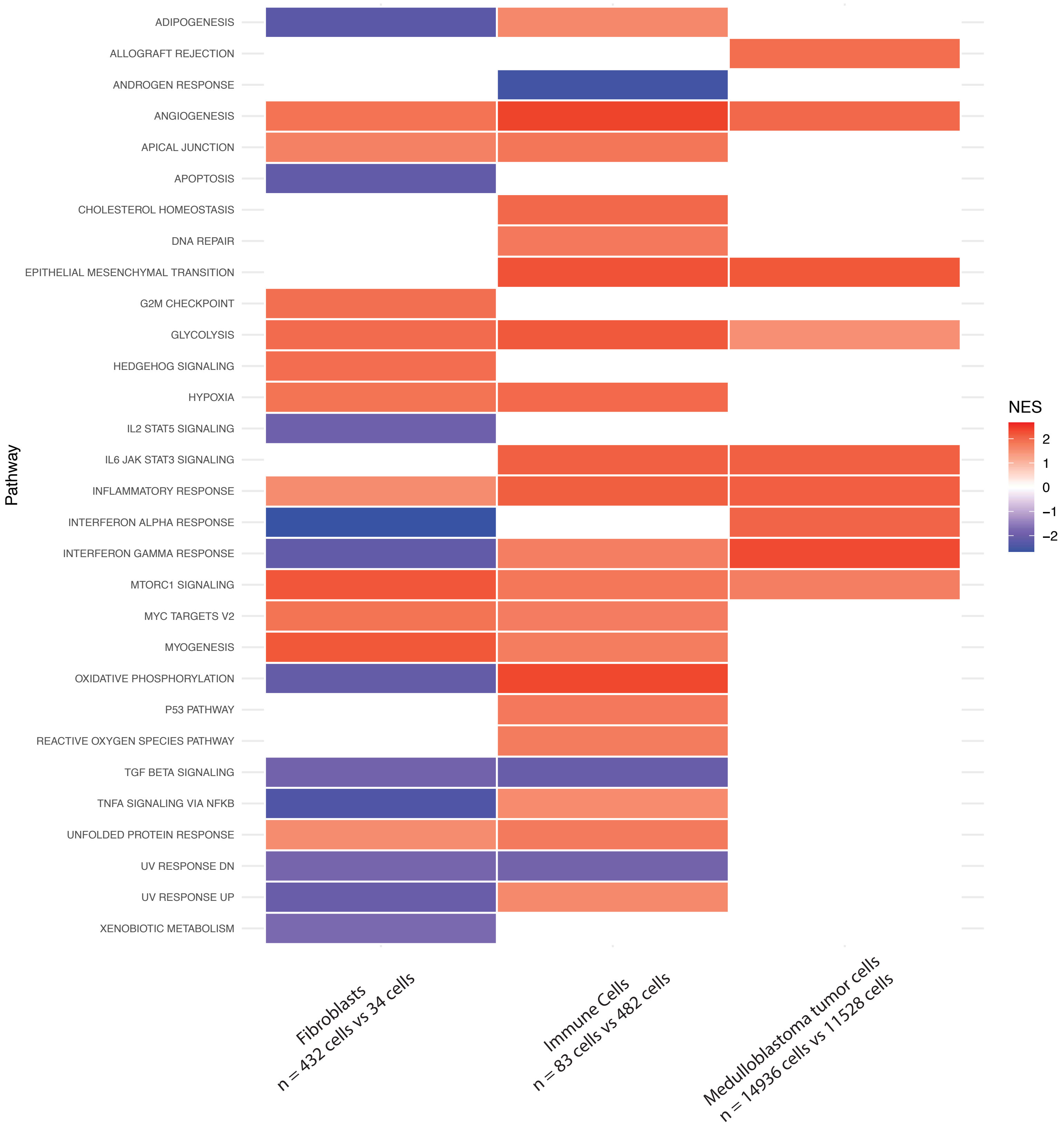

Supplementary Figure 4. MSigDB Hallmark analysis for the medulloblastoma sample. Heatmap of up- or down-regulated MSigDB Hallmarks across the three cell types identified in medulloblastoma sample, comparing day 14 cell type transcriptional profiles to day 0 transcriptional profiles. Normalized enrichment scores were generated, along with permutation-based adjusted p values for each pathway by cell type. Cell types that showed significant (Benjamini-Hochberg adjusted p values < 0.05) absolute normalized enrichment values > 1.5 were displayed, with the heatmap color corresponding to the normalized enrichment scores seen in the legend. Nonsignificant pathways are left blank. Number of cells in each cell type for day 0 and day 14 listed at bottom below cell type name. (NES = normalized enrichment score, MYC targets V2 = MYC targets, variant 2, DN = down)
